# Development of an array of molecular tools for the identification of khapra beetle (*Trogoderma granarium*), a destructive beetle of stored food products

**DOI:** 10.1038/s41598-023-29842-z

**Published:** 2023-02-27

**Authors:** Yunke Wu, Michael J. Domingue, Alana R. McGraw, Kendra A. Vieira, Marjorie Z. Palmeri, Scott W. Myers

**Affiliations:** 1 United States Department of Agriculture, Animal and Plant Health Inspection Services, Plant Protection and Quarantine, Science and Technology, Forest Pest Methods Laboratory, 1398 West Truck Road, Buzzards Bay, MA 02542 USA; 2 Department of Ecology and Evolutionary Biology, Cornell University, Ithaca, NY 14853 USA; 3 Department of Entomology, Kansas State University, Manhattan, KS 66502 USA; 4 Department of Environmental Conservation, University of Massachusetts, Amherst, Amherst, MA 01003 USA

**Keywords:** Invasive species, Entomology

## Abstract

*Trogoderma granarium* Everts, the khapra beetle, native to the Indian subcontinent, is one of the world’s most destructive pests of stored food products. Early detection of this pest facilitates prompt response towards the invasion and prevents the need for costly eradication efforts. Such detection requires proper identification of *T. granarium*, which morphologically resembles some more frequently encountered, non-quarantine congeners. All life stages of these species are difficult to distinguish using morphological characters. Additionally, biosurveillance trapping can result in the capture of large numbers of specimens awaiting identification. To address these issues, we aim to develop an array of molecular tools to rapidly and accurately identify *T. granarium* among non-target species. Our crude, cheap DNA extraction method performed well for *Trogoderma* spp. and is suitable for downstream analyses including sequencing and real-time PCR (qPCR). We developed a simple quick assay usingrestriction fragment length polymorphism to distinguish between *T. granarium* and the closely related, congeneric *T. variabile* Ballion and *T. inclusum* LeConte. Based on newly generated and published mitochondrial sequence data, we developed a new multiplex TaqMan qPCR assay for *T. granarium* with improved efficiency and sensitivity over existing qPCR assays. These new tools benefit regulatory agencies and the stored food products industry by providing cost- and time-effective solutions to enhance the identification of *T. granarium* from related species. They can be added to the existing pest detection toolbox. The selection of which method to use would depend on the intended application.

## Introduction

*Trogoderma granarium* Everts (Coleoptera: Dermestidae), the khapra beetle, is one of most devastating pest of a wide variety of stored food products, including cereals, grains, and many types of other commodities^1–4^. Its feeding and developmental activities can damage and contaminate up to 70% of the product and render the product unconsumable for human, causing hundreds of millions of dollars in damage^5^. It originates from the Indian subcontinent and has spread both regionally and worldwide (Mediterranean, Middle East, Africa, and North America) to areas with similar climates, including a temporary introgression into the warm, arid southwestern portion of the United States half a century ago^5,6^. Total eradication of the species from the region required substantial time and expense. For example, it took 13 years (1953–1966) and cost over 100 million in today’s dollar value for the successful eradication in California^6^. Furthermore, while it is presumed that *T. granarium* will perform better in warmer conditions, it also has the potential to cause severe problems in cooler climate areas within the confines of large food production and storage facilities that have sustained controlled environments. Such threatening infestations have been detected in warehouses of California, Pennsylvania, and Texas^7^. Because of this history of damage to the infrastructure of global food production systems, countries with established populations of this pest face export restrictions and regulation, while many countries without known populations have developed strict regulatory procedures regarding its management^6,8,9^. For example, *T. granarium* has been listed as an A2 quarantine pest by the European and Mediterranean Plant Protection Organization (EPPO)^10^.

**Figure 1:**
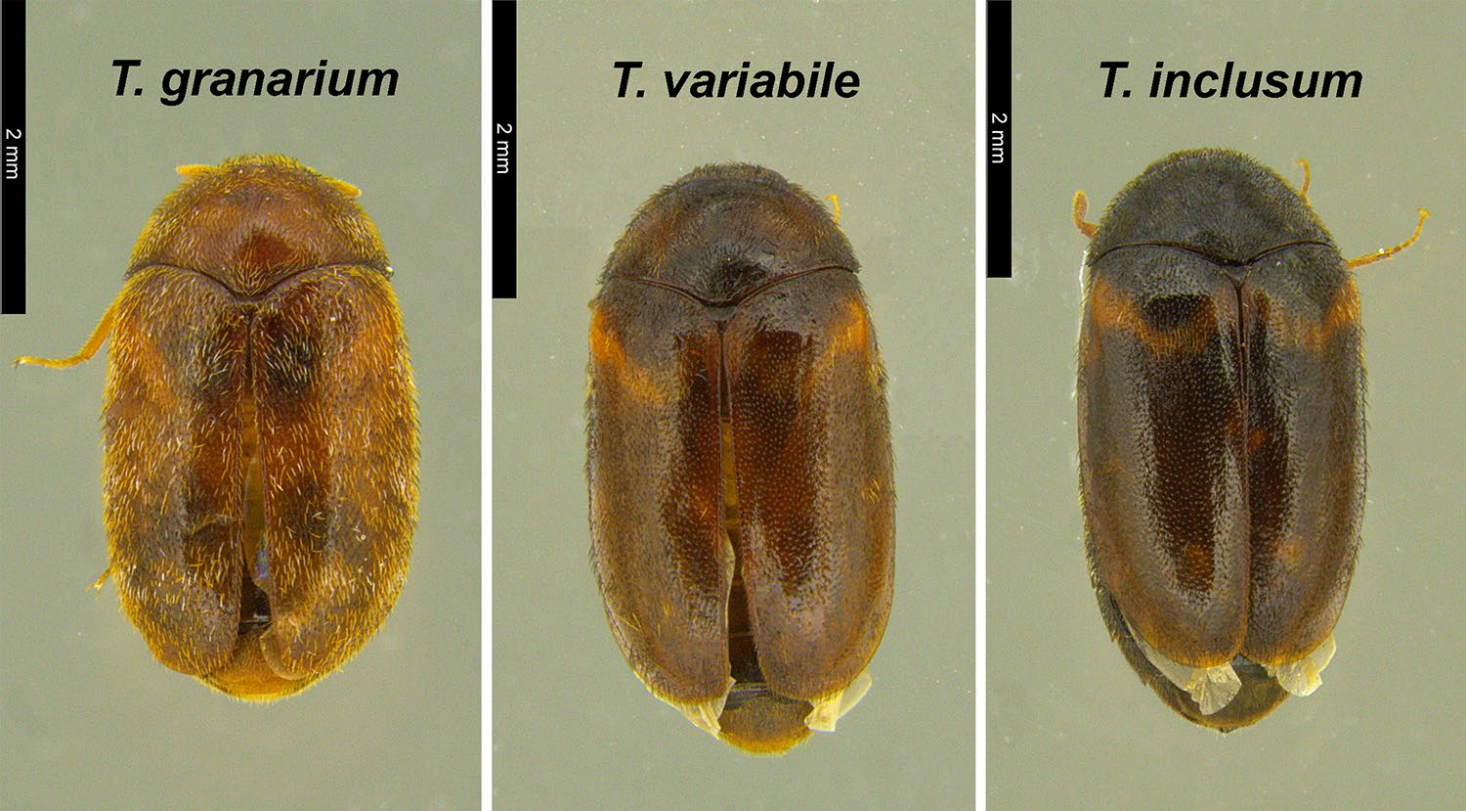
Dorsal view of the khapra beetle (*Trogoderma granarium*), warehouse beetle (*T. variabile*), and larger cabinet beetle (*T. inclusum*). Photo credit: Michael Martinson.

Early detection is a critical step to prevent the invasion of *T. granarium*. Such detection requires the use of biosurveillance traps^11^ or the inspection of commodities at ports of entry or other locations where *T. granarium* could cause substantial harm. Regardless of the method, a critical aspect of the process is the proper identification of the species. Another two congeneric species, the warehouse beetle (*T. variabile* Ballion) and larger cabinet beetle (*T. inclusum* LeConte) (Fig. 1), also affect stored food products in production and storage facilities. *Trogoderma variabile* is one of the most prevalent and damaging dermestids distributed worldwide, and *T. inclusum*, albeit less of a threat to stored food products, can co-occur with the former species^12^. They are both established in North America and are the most common *Trogoderma* spp. found in detection surveys for khapra beetle^13–15^*.* Although adult *T. granarium* are flightless^16^ and thus can be distinguished from the latter two species based upon assessing flight capability^17^, the use of morphological characteristics to distinguish among adults in this group requires microscope and trained expertise to implement in a program setting^18^, and dissection of genitalia is needed to confirm species-level determinations. In addition, it is often impossible to confidently identify larvae, especially when specimen quality has degraded or there is insufficient number of specimens to account for intraspecific variations. On the other hand, larvae are the predominant life stage encountered during inspection activities, particularly for *T. granarium*, because it spends most of its life as larva and can enter a prolonged facultative diapause at this stage.

Given the economic significance of khapra beetle and the need for prompt and accurate identification of suspected detections, several molecular assays have been developed based on real-time PCR (qPCR)^19,20^ and loop-mediated isothermal amplification (LAMP)^21^ to circumvent the challenges of using morphology. Molecular assay has been used to survey *Trogoderma* spp. in Spanish mills and warehouses^22^. The Australian government is also examining the use of these assays as identification tools for *T. granarium* and as a detection method of the species in environmental DNA (eDNA) samples^23^, where assay sensitivity is critical for successful application. For the existing Olson et al. qPCR assay^19^ (hereafter referred to as the Olson assay) and the Furui et al. qPCR assay^20^ (hereafter referred to as the Furui assay), assay sensitivity (measured as the limit of detection, LOD) is estimated at 100 copies/µl and 10 copies/µl, respectively^23^. The LAMP assay may have a lower sensitivity and failed with eDNA samples^21,23^.

Although existing qPCR assays performed within expected validation parameters for qualitative detection of tissue-derived *T. granarium* DNA, there are issues affecting their applications when target quantification is necessary. Amplification efficiency of the Olson assay on the same qPCR instrument varies widely between 72 and 93%^23^, which can be attributed to non-optimal primer design that includes primer self-complementarity and large discrepancy between the melting temperatures of forward and reverse primer. The Furui assay seems to have a higher amplification efficiency at 83.9–91%^23^. Ideal efficiency should be between 90 and 110% and better approaching 100%^24,25^. Less efficient qPCR leads to larger Ct (threshold cycle) that can confound result interpretation.

Besides the *T. granarium*-specific assay, both the Olson assay and the Furui assay designed a second, more general assay that provides a broad-scope identification to the genus *Trogoderma* or the family Dermestidae. Only when both the specific and the general assay are positive, can an identification of khapra beetle be made with confidence^19^. The general assay also serves an important role for DNA quality control, because a sole negative result of the specific assay suggests either a sample not being khapra beetle or a failed DNA extraction. But a positive result of the respective general assay from the same sample rules out the latter scenario. However, neither the Olson assay nor the Furui assay include multiplexing, meaning that the specific and general assay need to be run separately, which doubles the usage of reagents and increases processing time compared to multiplex qPCRs. Additionally, current designs of the general assays produce unusually large amplicons (> 400 bp), which significantly lower amplification efficiency^26^ and can lead to inconclusive identification^19^. Larger amplicons also have a lower probability of remaining intact in degraded specimens or eDNA samples.

In this study, we aim to introduce multiple new molecular tools to enhance the capacity of *T. granarium* detection, including (1) a validated inexpensive DNA extraction method that provides a cost-effective solution for obtaining DNA from a large quantity of individual specimens; (2) a PCR-based restriction fragment length polymorphism (RFLP) assay that can quickly diagnose colonies of *T. granarium*, *T. variabile*, and *T. inclusum* maintained in the United States, with the potential to be applied to field-collected specimens; (3) the first multiplex TaqMan qPCR assay that demonstrates high efficiency and sensitivity for *T. granarium* while addressing issues with existing qPCR assays. The new multiplex assay can be used for qualitative and quantitative applications and likely suitable for eDNA analysis. Integration of these tools into the khapra beetle detection toolbox—comprising inspection and trapping, dissection, and molecular assays—would benefit national and international plant protection organizations by improving the response efficiency for potential introductions of khapra beetle.

## Results

### ProtK DNA extraction

The crude DNA extraction method required a handling time of less than 1 min per specimen. For each insect, the half body processed with the ProtK method yielded approximately 60% less DNA than the other half processed with the DNeasy Blood & Tissue Kit. Average DNA yield from the former method was 0.94 ng/µl (0.30–2.07 ng/µl) compared to 2.51 ng/µl (0.52–5.36 ng/µl) from the latter method. Despite lower DNA yield, all 32 ProtK extracts produced strong positive PCR amplification. Electrophoresis showed that PCR products running side-by-side with those from the DNeasy Blood & Tissue Kit had comparable or only slightly weaker brightness (Supplementary Information). Sanger DNA sequencing of PCR products from the ProtK method generated high quality data, which were used for subsequent assay development. For the 1200 larval and adult colony specimens, ProtK extraction success rate was 96.9% (1163/1200).

### PCR–RFLP assay for Trogoderma spp

**Figure 2:**
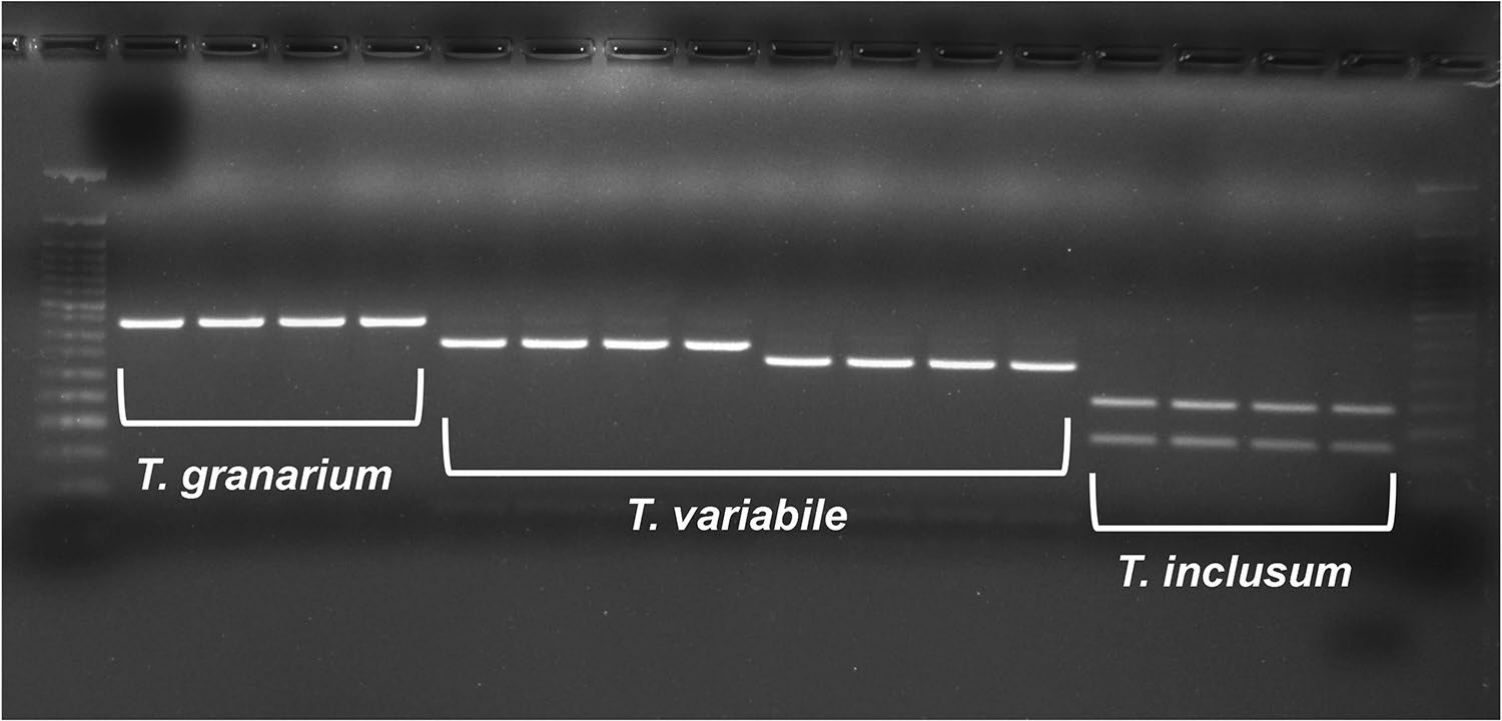
Digestive patterns of *Trogoderma granarium*, *T. variabile*, and *T. inclusum* for the mitochondrial NJ/MD region with the NlaIII endonuclease. There is no enzyme recognition site in *T. granarium*, one or two sites in *T. variabile* near the rear end or both ends, and one site in *T. inclusum* towards the middle of the sequence. Digested fragments < 100 bp are very faint. Left and right ladder: 50 bp DNA Ladder (New England BioLabs).

Unlike the universal COI primers LCO1490 and HCO2198, the modified primer pair amplified 100% *Trogoderma* spp. samples and resulted in high quality sequence data (GenBank accessions OQ358883–OQ358900). Sample identities were confirmed by querying against the Barcode of Life Data System (BOLD^27^). Then we aligned newly generated NJ/MD sequences from the three species (GenBank accessions OQ358902–OQ358923) along with the five partial mitochondrial genomes from GenBank. Several commercially available restriction enzymes were analyzed. We chose NlaIII because it generated distinct digestive patterns among the three tested species and further separated the two colonies of *T. variabile* (WBLAB-Tv vs. EKS316-Tv). Those published mitochondrial genome sequences, despite their different geographic origins, shared the same digestive pattern with the colonies. The NJ/MD sequence of *T. granarium* did not possess any NlaIII recognition site and thus remained intact after digestion, whereas sequences from *T. variabile* and *T. inclusum* were digested near the end(s) and towards the middle, respectively. Since DNA fragments smaller than 100 bp were very faint or invisible on the 3% agarose gel, the electrophoresis generated a single DNA band (437 bp) for *T. granarium*, a smaller single band (376 or 336 bp) for *T. variabile*, and two small bands (181 bp + 256 bp) for *T. inclusum* (Fig. 2). After applying the assay to the 1200 *Trogoderma* specimens extracted by the ProtK method, we were able to diagnose them into 333 T*. granarium* and 830 T*. inclusum*; 37 failed PCR. For the 34 specimens randomly selected for DNA barcoding, identification matched 100% between PCR–RFLP and sequencing results.

### Multiplex qPCR assay for khapra beetle

**Figure 3:**
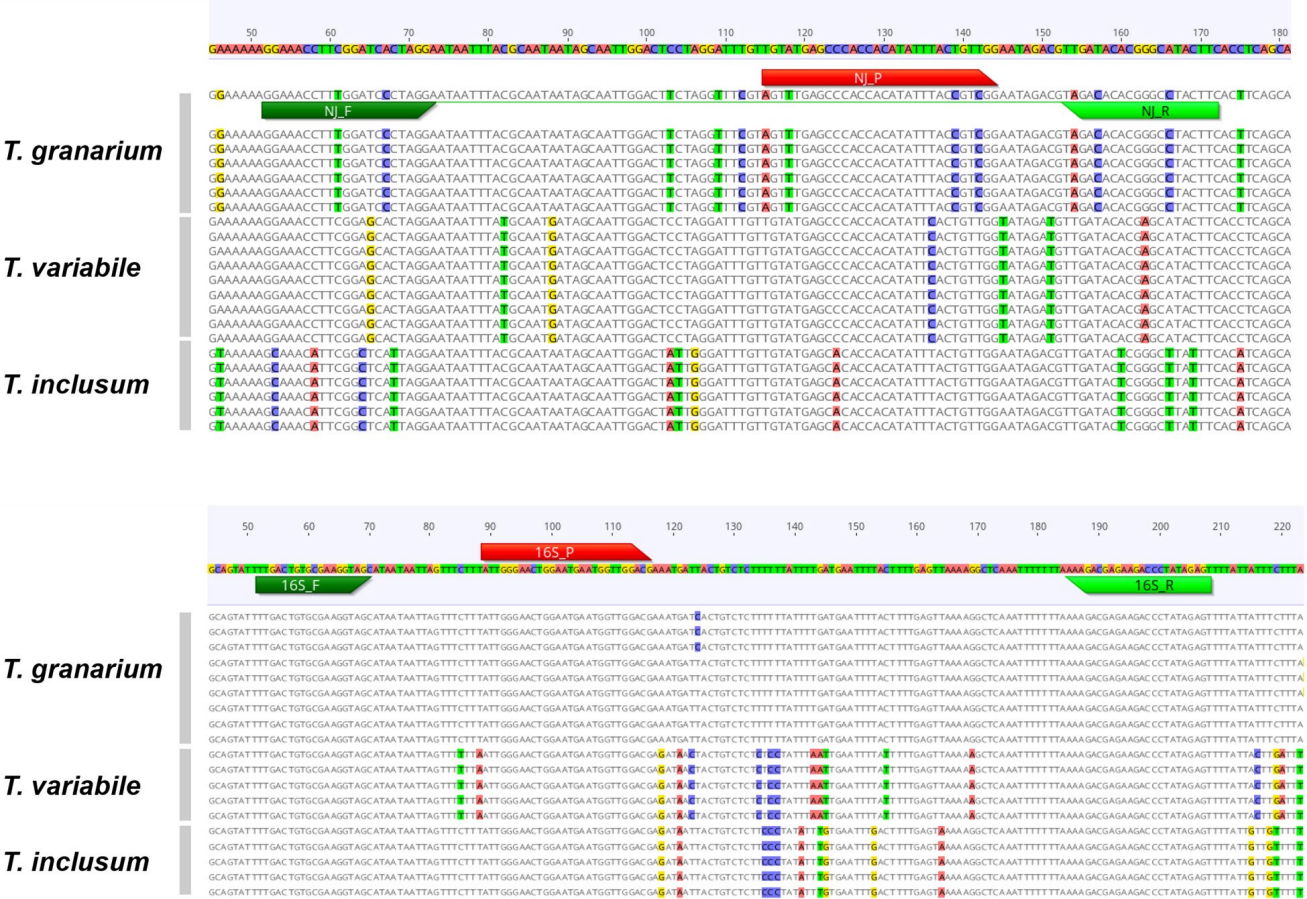
Sequence alignment showing primer and probe sequences of the *T. granarium* specific assay (top) and the general assay (bottom). Oligo sequences are unique to *T. granarium* in the specific assay but conserved across the three species in the general assay.

The newly generated NJ/MD sequence data for developing the specific assay exhibited large interspecific genetic distances (uncorrected p-distance 15.4–16.6%). However, intraspecific differences were minor among colonies as well as between colonies and published mitochondrial genomes (*T. granarium*, 0–0.7%; *T. variabile*, 0–1.2%; *T. inclusum*, 0%), suggesting that colony specimens were good representations of their respective species. A primer–probe combination unique to *T. granarium* was identified near the 5′ end of the NJ/MD sequences (Table 3). Primer and probe sequences differed from the other two congeners by 3–6 SNPs (Single Nucleotide Polymorphisms) and thus off-target amplification should be prevented or reduced (Fig. 3). BLAST specificity analysis found very few hits for the primer and probe sequences among published insect sequence data: only 11 hits were found with ≤ 2 mismatches from the primer pair, and 15 hits with ≤ 1 mismatch from the probe, 12 of which belonged to *T. granarium* itself (the other three were a leaf beetle, a longhorned beetle, and a false darkling beetle). The only overlap between the two sets of hits was *T. granarium*, supporting high assay specificity. After running the qPCR assay on those colony specimens, all *T. granarium* produced positive results in the specific assay, which were negative for the 76 *T. variabile* and 68 *T. inclusum* specimens (i.e., no off-target amplification). In contrast, all three species produced positive results in the general assay (Supplementary Information).

Regarding the development of the general assay, 16S sequence data obtained from GenBank again showed considerable divergence among the three species of *Trogoderma* (uncorrected p-distance 12.2–16.1%). Two published sequences under the name of *T. granarium*, KJ930480, and KJ930496, were probably mislabeled, as they were highly similar or identical to *T. ornatum* and *T. glabrum*, respectively. Despite large genetic divergences, we identified a highly conserved region near the 5′ end of the sequences to design primers and probes (Fig. 3). BLAST analysis for the general assay found 951 hits among published insect sequence data that were with ≤ 2 mismatches from the primer pair and 290 hits with ≤ 1 mismatch from the probe. The two sets of hits overlapped only for the genus of *Trogoderma* and a stag beetle (*Sinodendron cylindricum*) native to Europe. Our result indicated that six additional *Trogoderma* spp. likely can be detected by the general assay, including *T. anthrenoides*, *T. glabrum*, *T. grassmani*, *T. ornatum*, *T. simplex*, and *T. teukton*, all of which shared identical primer sequences with the three tested species and had at most one mismatch in the probe sequence (mismatch was outside the last five nucleotide position of the 3′ end).

The multiplex qPCR performed well with all serial dilutions of *T. granarium* DNA templates (Supplementary Information). Standard curves revealed high repeatability as technical replicates had small variations: Ct standard deviation ≤ 0.50 for the specific assay and ≤ 0.57 for the general assay. For each dilution, differences of Ct values between multiplexing vs. individual mode were about one unit or less (maximum difference = 1.27), suggesting little interference between the two assays during multiplexing. Regardless of running mode, assay linearity had a very high fit (all R^2^ ≥ 0.995). The LOD was determined to be 5 DNA copies/µl because all technical replicates readily amplified (specific assay: mean Ct = 36.00; general assay: mean Ct = 36.31). Further lowering DNA copies could compromise assay repeatability. The determined LOD was already close to the theoretical value of the most sensitive LOD (i.e., 3 DNA copies per reaction^28^). Amplification efficiency reached 97.9% for the specific assay and 96.9% for the general assay when running in multiplex. When running individually, the amplification efficiency was 101.5% and 95.8%, respectively (Supplementary Information), which are well within the desired range of 90–110% and higher than that of existing khapra beetle qPCR assays.

## Discussion

To prevent the invasion of khapra beetle, we developed several molecular tools, ranging from inexpensive DNA extraction method to PCR–RFLP and qPCR assay, which together will enhance diagnostics of its larval and adult samples. These tools are particularly useful for biosurveillance trapping surveys or inspections that may result in the collection of hundreds of closely related, non-regulated species that are difficult and time-consuming to identify through morphology. Detection of khapra beetle outside of its current range typically leads to prompt treatment and/or delimiting surveys to ensure that the infestation is contained and that any populations are eradicated. The enhanced identification capacity would reduce the time needed to initiate such critical responses. The exclusion of khapra beetle is particularly critical for grain-producing countries as detections and potential establishment can threaten export markets.

Successful DNA extraction is fundamental to molecular diagnostics. When processing a handful of samples, commercial kits such as the Qiagen DNeasy Blood & Tissue Kit provides a reliable option. However, the cost (~ $3 per sample) and time needed for benchwork increase rapidly if there are hundreds and thousands of samples awaiting identification, such as bulk sample collections of insects. In cases with large numbers of samples, batch-processing has been adopted to reduce cost and workload^29–31^, but there are also circumstances when samples are processed individually. The ProtK method developed here can be a cost-effective solution to extract DNA from a large number of samples. Reagents used here are inexpensive, as the total cost per sample is $0.04 or less. The required benchwork only involves cutting and placing specimens into the extraction buffer, with the overnight incubation taking up most of the processing time. Future studies can assess the effect of incubation time on DNA extraction success. Highest efficiency can be achieved by using 1.2 ml 96-well microplate cluster tubes with a multi-channel pipette.

Side-by-side comparison between ProtK and Qiagen extractions shows that both methods produced positive PCR amplification from laboratory-reared specimens, although the average DNA yield was lower using the former method, where tissue was not homogenized. Chopping the insect into small pieces will likely increase DNA yield. It also should be noted that DNA yield in the ProtK extracts could be lower than the fluorometer readings due to the potential presence of protein and other contaminants, since no centrifugation or filtration was involved. However, neither issue had a negative impact on subsequent PCR or qPCR. In a separate study that explored the outcome of direct competition between *T. granarium* and *T. inclusum* in stored food products settings (also see below^32^), we used the ProtK method to extract DNA from 1200 larval and adult colony specimens and the success rate was 96.9%. qPCR analysis of those samples produced Ct values around 15–25, equivalent to target DNA concentrations in the range of 10^4^–10^7^ copies/µl according to the standard curves, which are far above the assay LOD (5 copies/µl).

Compared to other crude DNA extraction methods, such as the HotSHOT technique, the ProtK method uses proteinase K instead of NaOH to digest protein and remove contaminants, whereas NaOH not only dissolves organic matters but denatures DNA into the single-stranded form^33^. However, it has been reported that NaOH can harm the quality of DNA extracted from insect larvae^34^. Assay failure resulting from the HotSHOT DNA extraction method can be close to 50% in molecular diagnostics of khapra beetle^21^. Other cheap extraction methods like sodium dodecyl sulfate (SDS) and cetyltrimethyl ammonium bromide (CTAB) are time-consuming and require a fume hood to operate^35^. There are also commercially available crude extraction kits such as the QuickExtract solution, but it costs over $7 per sample and its ingredients are proprietary (i.e., trade secret). Therefore, the ProtK method provides an effective alternative to existing crude DNA extraction protocols.

Diagnostic assays based on restriction fragment length polymorphism (RFLP), which was first introduced in the late 1970s and early 1980s^36^, gained much popularity but in recent years appeared to give way to sequencing, qPCR, and other advanced molecular techniques. However, RFLP based on PCR products is still proven to be a powerful tool for species identification in economically and medically important taxa^37,38^, because it is relatively simple in design and results are easy to interpret, as different target species exhibit distinct digestive patterns allowing for unambiguous identification. The PCR–RFLP assay developed here confirmed its usefulness in distinguishing between colonies of *T. granarium*, *T. variabile*, and *T. inclusum* maintained in the United States. The three species can be readily separated through electrophoresis after digestion. In the aforementioned study^32^ that focused on direct competition between *T. granarium* and *T. inclusum*, which were housed together in the same containers for weeks, the PCR–RFLP assay effectively identified 1163 surviving larvae and adults to either species out of 1200 tested insects. Those identifications made by the PCR–RFLP method were further validated through sequencing. The PCR–RFLP method has its advantage over sequencing or qPCR for identifying a large number of samples given its relatively low cost (~ $0.3 per specimen). It is also less prone to be affected by contamination as long as the target DNA is abundant, which will dominate over non-target DNA during the PCR process. In the case of the direct competition study^32^, surviving insects can have the competitor’s DNA attached to its body surface or even in its digestive system. Nonetheless, the PCR–RFLP assay correctly identified the surviving insect, while the presence of the competitor’s DNA was indeed detected by our qPCR assay (data not shown).

The cornerstone of PCR–RFLP assay, endonuclease recognition sites, is also its Achilles' heel. When mutations alter DNA bases of the recognition site or create new sites in untested samples, these changes need to be accounted for when analyzing digestive patterns^36^. Although intraspecific divergences in the genus *Trogoderma* are around 1% or less according to published genetic data, and GenBank sequences of *T. granarium* and *T. variabile* with different geographic origins also conform to the anticipated digestive patterns, new variations may still be present in populations not sampled. Consequently, it is necessary to test additional, field-collected populations to assess the performance of the assay and incorporate possible variations. A second restriction enzymes, such as the commonly used Hinfl, can be included along with NlaIII to generate a double digest pattern, which tends to have a higher resolution for species identification. Nevertheless, the PCR–RFLP assay in its current form could steadily diagnose the three species *Trogoderma* maintained at colonies in the United States.

The newly developed TaqMan qPCR assay aims to provide a high level of efficiency and sensitivity for detecting khapra beetle DNA. All tested *T. granarium* produced positive amplification in both the specific and the general assay, whereas the non-target *T. variabile* and *T. inclusum* were only positive for the general assay. Assay sensitivity at 5 copies/µl is by far the highest. Importantly, its multiplexing capacity significantly saves processing time and reduces reagent cost. For the Olson assay and the Furui assay, it is possible to only run their specific assays, but there are risks of encountering false positive (off-target amplification) or false negative (failed DNA extraction), although subsequent sequencing of the qPCR product can eliminate false positives^23^.

The BLAST analysis supports that the specific assay is indeed specific to *T. granarium*, as we did not find close matches to the primer–probe combination in other published insect sequences. For congeneric taxa with no or limited sequence data in GenBank, the possibility of a false positive should remain low, because mitochondrial sequence divergences between different species of *Trogoderma* are considerably large, ranging between 10 and 26%^19^, which is in line with our data. Based on this level of divergence, it is reasonable to expect > 2 SNPs in either primer sequence (20 and 22 bp, respectively) and > 3 SNPs in the probe sequence (30 bp) among other *Trogoderma* spp. Although our study did not include *T. glabrum* due to the lack of specimens, which was reported as the most closely related pest species to *T. granarium*, the included *T. inclusum* and *T. variabile* are khapra beetle’s next close relatives^19^. More importantly, the latter two species are by far the most prevalent *Trogoderma* spp. found in facilities and shipment of stored food products in the United States^12,15^. They differ from *T. granarium* by at least 3 SNPs in the forward primer, reverse primer, and probe sequence, respectively. In comparison, the Olson assay achieved its specificity only through the primer sequences unique to *T. granarium*, whereas the probe sequence is conserved across the genus.

Under rare circumstances when off-target amplification does occur in the new multiplex qPCR assay, two measures can ensure that false positives are properly identified. First, because both the specific and the general assay are based on mitochondrial genes, given similar amplification efficiencies (96.9% and 97.9%, respectively), their Ct values from the same *T. granarium* sample are expected to be nearly identical. In fact, Ct values between the two assays are within 0.5 units for *T. granarium* samples tested here. Therefore, a large discrepancy between Ct values of the two assays, including one assay being positive and the other being negative, would indicate that the specimen is not a khapra beetle. Secondly, our analysis showed that when the target DNA is at 5 copies/µl (10 copies per reaction), Ct value of either assay was around 36. In theory, the most sensitive LOD possible is 3 DNA copies per reaction^28^. Extrapolating from the standard curves, we consider Ct values > 39 no longer reliable, especially in the absence of PCR inhibitors which can be reduced by sample dilution^25^. On the other hand, the likelihood of encountering a false negative for the specific assay is probably also low. False negatives would happen when an untested *T. granarium* population/individual exhibits novel SNPs that lower qPCR efficiency. However, the Olson assay study that analyzed *T. granarium* from multiple geographic sources worldwide found < 0.5% intraspecific divergence^19^, suggesting conserved mitochondrial sequence in this species. Indeed, published GenBank sequences with geographic origins different from our colonies (e.g., India, Saudi Arabia, Syria, United Arab Emirates, Turkey) all have identical primer/probe sites, indicating high likelihood of successful qPCR amplification. Nevertheless, testing additional wild populations of *T. granarium* provides an avenue for future research.

The general assay serves a dual purpose of assessing DNA extraction success and preliminary detection of *Trogoderma* spp. Specimens other than khapra beetle detected by the general assay indicates a successful DNA extraction, which reinforces a negative result of the specific assay as being truly negative. In addition to the three species tested here, six other *Trogoderma* spp. will likely be detected by the general assay, suggesting a broader generality of the assay to cover congeneric taxa, some of which are also considered pests that are worthy of the detection effort^39^. Knowing that a specimen belongs to the genus *Trogoderma* will inform regulatory agencies about potential damage. Species-level identification can be subsequently obtained through DNA-barcoding using the modified COI primers provided in this study. It must be noted that the general assay should not be used as a sole evidence for detection of the *Trogoderma* spp. until further validation is made with specimens from closely related genera.

We demonstrated high sensitivity of the new multiplex qPCR assay, which is critical for detecting *T. granarium* against a background of non-target pest beetles of stored grain products. Additionally, the presence of *T. granarium* can be traced down to a small amount of DNA released into the environment through shed cells, exuviae, secretion, etc. The existing and new qPCR assays may form powerful detection tools when used in conjunction with the eDNA technique, which is particularly suitable for biosurveillance responses such as inspections of warehouses or cargo. However, during empirical applications it is recommended to re-evaluate the LOD, as the limit determined with laboratory-prepared DNA templates can be different from that generated by field samples due to the presence of PCR inhibition and interference^24^.

## Conclusion

Traditional methods might not be able to accurately detect and identify khapra beetle due to conserved morphology between closely related species, some of which could be more frequently encountered than khapra beetle. Here we presented molecular tools to diagnose various life stages of khapra beetle under different circumstances. The crude DNA extraction method serves as an inexpensive solution for analyzing a large quantity of beetle or larval samples. The PCR–RFLP assay proves its usefulness in effective identification of colony insects belonging to the genus *Trogoderma* and has the potential to be applied to field-collected specimens. The new qPCR assay not only increased processing efficiency through multiplexing but demonstrated improved characteristics over existing assays. It provides the highest level of diagnostic sensitivity for detecting target DNA. Together with existing assays in the current pest detection toolbox, these tools can be implemented individually or in combination to maximize diagnostic efficiency while keeping the cost low, making them adaptable to large-scale screening efforts. Tool selection would depend on the intended application. Prompt and accurate detection of *T. granarium* will facilitate international grain trade and contribute to safeguarding the stored food products industry.

## Materials and methods

### Beetle sampling

Specimens of *T. granarium*, *T. variabile*, and *T. inclusum* were obtained from seven colonies maintained at Forest Pest Methods Laboratory (US Department of Agriculture APHIS PPQ) and Center for Grain and Animal Health Research (US Department of Agriculture ARS) (Table 1). The *T. granarium* colony was collected from Pakistan in 2011 and represents the only colony in North America. To our best knowledge, no other institute or agencies maintain colonies of *Trogoderma* in the United States. They are all reared under similar conditions using ~ 1 L glass jars in separate rooms on a 4:1:1 ratio by volume of ground dog food (Newman's Own Adult Dog Formula, Aptos, CA), raw wheat germ (Mennel Milling, Fostoria, OH), and rolled oats (Bob’s Red Mill, Milwaukie, OR). Colony jars were maintained in an environmental growth chamber (Percival Corporation, Perry, IA) at 32 °C in continuous darkness.

**Table 1:** Specimens used for development of molecular assays.

Species	Facility	Colony ID	Collection location	Collection date
*Trogoderma granarium*	Forest Pest Methods Laboratory, APHIS	n/a	Faisalabad, Pakistan	2011
*Trogoderma variabile*	Center for Grain and Animal Health Research, ARS	WBLAB-Tv	Unknown	Unknown
	Center for Grain and Animal Health Research, ARS	EKS316-Tv	Feed mill in Emporia, Kansas	March, 2016
*Trogoderma inclusum*	Center for Grain and Animal Health Research, ARS	FieldAug2012-Ti	North-central Kansas	August, 2012
	Center for Grain and Animal Health Research, ARS	KS712-Ti	Flour mill in Stafford County, Kansas	July, 2012
	Center for Grain and Animal Health Research, ARS	AR712-Ti	Rice mill in Arkansas	July, 2012
	Center for Grain and Animal Health Research, ARS	109-Ti	Unknown	Unknown

### A crude DNA extraction method

We developed a simple DNA extraction method, termed the “ProtK method”, that utilizes one extraction buffer comprised of proteinase K, TERGITOL NP-40 (a surfactant), and TE buffer. We prepared 0.1% TERGITOL in 1X TE buffer and added proteinase K powder to a concentration of 0.05 mg/ml. Each adult beetle or larva was cut into two or three pieces, submerged in 500 µl of the prepared extraction buffer, and incubated at 37 °C overnight. Genomic DNA was gradually released into the buffer. The proteinase K was deactivated the next morning by heating the DNA extract to 75 °C for 30 min on a heat block. The DNA extract was then cooled and stored at − 20 °C for subsequent use. To compare DNA quality obtained from the ProtK method to that from the widely used DNeasy Blood & Tissue Kit (QIAGEN, Germantown, MD, USA), we obtained 16 adults and 16 larvae of all three species from the colonies. Each specimen was cut longitudinally into two halves and one half was extracted using the ProtK method while the other was extracted using the DNeasy Blood & Tissue Kit. Extracted DNA was quantified with a Qubit 2.0 fluorometer. PCR products resulting from the two methods were run side-by-side on 3% agarose gel pre-stained with SYBR Safe (Thermo Fisher Scientific, Waltham, MA, USA). We further applied the ProtK extraction method to 1200 *Trogoderma* larval and adult specimens from the colonies. Those specimens were used for a separate study^32^ and consisted of live adults and larvae reared under similar conditions as described above. Positive PCR amplification from samples while no amplification from negative controls would indicate successful DNA extraction.

### Development of PCR–RFLP assay

To confirm species identity of the USDA colonies, four beetles per colony were selected for DNA barcoding based on the mitochondrial COI region. Previous reports indicated that the universal COI primers LCO1490 and HCO2198^40^ worked poorly with the genus^19^. Therefore, we obtained five partial mitochondrial genomes from GenBank (*T. granarium*: LC386208–09, MG011536, MT113335; *T. variabile*: MG011537, MH922966) and modified the universal primers to be able to readily amplify *Trogoderma* spp. (Table 2). Cycling conditions consisted of initial denaturation at 94 °C for 2 min, followed by 40 cycles of denaturation at 94 °C for 15 s, annealing at 52 °C for 30 s, extension at 72 °C for 1 min, and a final extension at 72 °C for 5 min.

**Table 2:** Primer sequences for DNA barcoding and the RFLP assay for *T. granarium*, *T. variabile*, and *T. inclusum*.

Forward/reverse	Primer sequence	Source
DNA barcoding		
TrogoCOI_F	TTTCYACAAACCACAAAGACATTGG	Modified from Ref^40^
TrogoCOI_R	TAAACTTCTGGGTGYCCRAAGAATCA	Modified from Ref^40^
RFLP assay		
TrogoNJ_F	TACATTCTAATYCTACCAGGATTTGG	Modified from Ref^41,42^
TrogoMD_R	ATGGCGAATATTGCTCCTAT	Modified from Ref^43^

After species identity was confirmed, a PCR–RFLP assay was developed based on a 437 bp mitochondrial fragment immediately downstream of the COI barcoding region (the “NJ/MD” region^41–43^). Forward and reverse primers were modified from earlier studies to specifically amplify the genus *Trogoderma* (Table 2). Cycling conditions consisted of initial denaturation at 93 °C for 3 min, followed by 35 cycles of denaturation at 93 °C for 15 s, annealing at 46 °C for 45 s, extension at 68 °C for 45 s, and a final extension at 68 °C for 7 min. Six to eight specimens per species were sequenced for the NJ/MD region. In silico restriction analysis was performed on the resulted DNA alignment in Geneious Prime 2021.1.1. We aimed to choose a restriction endonuclease that provides maximum distinct digestive results among the three species. The selected enzyme was ordered from New England Biolabs (Ipswich, MA) and digestion was performed according to the manufacturer’s protocol, except that the reaction volume was lowered to 15 µl of digest mix with 5 µl of PCR product. Digested PCR products were run on 3% agarose gel pre-stained with SYBR Safe. The PCR–RFLP assay was subsequently applied to the 1200 *Trogoderma* specimens^32^ extracted by the ProtK method for species identification. Thirty-four identified specimens were selected for sequencing to validate PCR–RFLP results.

### Development of multiplex qPCR assay

To detect *T. granarium* DNA, we developed a multiplex TaqMan qPCR that included a khapra beetle-specific assay and a more general assay, which serves a dual purpose of DNA quality control and broad-scope detection of the three *Trogoderma* spp. tested here (potentially other species of *Trogoderma* as well). Primers and probes were designed using Primer 3^44^ implemented in Geneious Prime. We followed the Geneious guideline that aims for an optimal primer T_m_ of 58–60 °C, a probe T_m_ of 65–70 °C, and an amplicon size range of 100–150 bp. The specific and general assay targeted two different mitochondrial genes: the former was developed based on the NJ/MD alignment generated in this study and the latter was based on the published 16S data^19^  (Table 3).

**Table 3:** Primers and probes of the multiplex qPCR assay used for detection of khapra beetle.

	Primer and probe sequence	T_m_ (°C)	Amplicon size
*T. granarium* specific assay			
Forward (NJ_F)	GGAAACCTTTGGATCCCTAGGA	59.1	121 bp
Reverse (NJ_R)	GAAGTAGGCCCGTGTGTCTA	58.8	
Probe (NJ_P)	AGTTTGAGCCCACCACATATTTACCGTCGG	68.4	
16S general assay			
Forward (16S_F)	TTGACTGTGCGAAGGTAGC	57.8	157 bp
Reverse (16S_R)	ACTCTATAGGGTCTTCTCGTCTT	58.8	
Probe (16S_P)	ATTGGGAACTGGAATGAATGGTTGGACG	65.7	

Potential oligo-dimer structures between multiplexing primers and probes were analyzed using Multiple Primer Analyzer (https://www.thermofisher.com/us/en/home/brands/thermo-scientific/molecular-biology/molecular-biology-learning-center/molecular-biology-resource-library/thermo-scientific-web-tools/multiple-primer-analyzer.html) from ThermoFisher Scientific (Waltham, MA, USA) and OligoAnalyzer Tool (https://www.idtdna.com/calc/analyzer) from Integrated DNA Technologies Inc. (Coralville, IA, USA), where primers and TaqMan probes were synthesized. Probes for the specific and general assay were labeled with the fluorophore 6-FAM and HEX, respectively, and quenched by Iowa Black FQ (IBFQ). Due to the extended length of the probes, an internal quencher ZEN was placed after the 9th base from the reporter dye on the 5′ end of the probe to enhance the quenching effect. To assess assay specificity, we blasted those DNA oligo sequences against published sequences in GenBank (as of March 18, 2022). Forward and reverse primer pairs were evaluated within the Class Insecta (taxid: 50,557) using the Primer-BLAST^45^ (https://www.ncbi.nlm.nih.gov/tools/primer-blast/) as implemented on the NCBI website. TaqMan probes were evaluated using the standard nucleotide BLAST (blastn) with search parameters adjusted for short input sequence within the Class Insecta for the first 500 hits. Only hits with ≤ 2 mismatches from the primer pair or with ≤ 1 mismatch from the probe sequence were recorded, as multiple mismatches in primers and probes can have a profound negative effect to amplification, especially when mismatches are located near the 3′ end of the sequence^46,47^.

The qPCR was performed on a StepOnePlus Real-Time PCR System (ThermoFisher Scientific). A 10 µl reaction mixture contained 5 µl of 2X QuantiTect Multiplex PCR NoROX master mix (Qiagen), 0.5 µl of primer/probe mixture with a final concentration of 500 nM for each primer and 100 nM for each probe, 2.5 µl of water, and 2 µl of DNA template. Cycling conditions consisted of an initial 95 °C for 15 min to activate the HotStartTaq DNA Polymerase, followed by 45 cycles of 2-step cycling of denaturation at 95 °C for 15 s and combined annealing and extension at 60 °C for 1.5 min. Ct value was determined by placing the fluorescence threshold at 10% of maximum fluorescence from positive controls^48^. We ran the multiplex assay with eight specimens of *T. granarium*, 76 T*. variabile*, and 68 T*. inclusum*.

To validate the qPCR assay, we created standard curves in both multiplexing and individual modes. Assay repeatability (intraassay variation), linearity (R^2^), sensitivity (LOD), and amplification efficiency were calculated. To prepare serially diluted DNA template, we first amplified the NJ/MD and 16S fragment of *T. granarium* using TrogoNJ_F and TrogoMD_R (Table 2) and 16S LR-J-12961 and 16S LR-N-13398^19^, respectively, because qPCR products are nested within these two fragments. Amplified PCR products were visualized on agarose gels by the Axygen Gel Documentation Systems (Corning, Glendale, AZ, USA) and then excised with the QIAquick Gel Extraction Kit (QIAGEN) following the manufacturer’s protocol. This step removed dNTP, excessive primers, and other non-target small DNA contained in the PCR reaction mix. Purified DNA templates were quantified by Qubit 2.0 fluorometer using the dsDNA BR Assay Kit (ThermoFisher Scientific). Template concentration was converted from ng/µl to copies/µl by the DNA Copy Number and Dilution Calculator (ThermoFisher Scientific, https://www.thermofisher.com/us/en/home/brands/thermo-scientific/molecular-biology/molecular-biology-learning-center/molecular-biology-resource-library/thermo-scientific-web-tools.html), given the product length of 437 bp of the NJ/MD and 469 bp of the 16S fragment. We prepared eight serial dilutions of 1:10 from 5 × 10^7^ copies/µl to 5 copies/µl per target by mixing the two DNA templates of equal amount. Each dilution was run with four technical replicates. Lastly, we determined the assay LOD following the MIQE guideline^49^ by requiring 100% probability of detection while maintaining standard curve linearity R^2^ > 0.98.

## Supplementary Information

Supplementary Information.

## Data Availability

Sequences were deposited in the NCBI GenBank database (https://ncbi.nlm.nih.gov/genbank) under accessions OQ358883–OQ358900 and OQ358902–OQ358923.
