# Supplementary material for: Development of an array of molecular tools for the identification of khapra beetle (Trogoderma granarium), a destructive beetle of stored food products
Source: Sci Rep. 2023 Feb 27;13:3327. doi: 10.1038/s41598-023-29842-z (PMC9971273; doi:10.1038/s41598-023-29842-z)

**Development of an array of molecular tools for identification of khapra beetle (*Trogoderma granarium*), a destructive beetle of stored food products**

Yunke Wu^1,2^, Michael J. Domingue^1,3^, Alana R. McGraw^1,3^, Kendra A. Vieira^1^, Marjorie Z. Palmeri^1,4^, Scott W. Myers^1^

^1^United States Department of Agriculture, Animal and Plant Health Inspection Services, Plant Protection and Quarantine, Science and Technology, Forest Pest Methods Laboratory, 1398 West Truck Road, Buzzards Bay, MA, 02542, USA

^2^Cornell University, Department of Ecology and Evolutionary Biology, Ithaca, NY 14853, USA

^3^Kansas State University, Department of Entomology, Manhattan, Kansas, 66502, USA

^4^Department of Environmental Conservation, University of Massachusetts, Amherst, Amherst, MA 01003, USA

Running title: New molecular diagnostic tools for identification of khapra beetle

Correspondence: Yunke Wu, [yunke.wu@usda.gov](mailto:yunke.wu@usda.gov)

# Supplementary

Figure S1. Side-by-side comparison of PCR products from the ProtK method and DNeasy Blood & Tissue Kit.


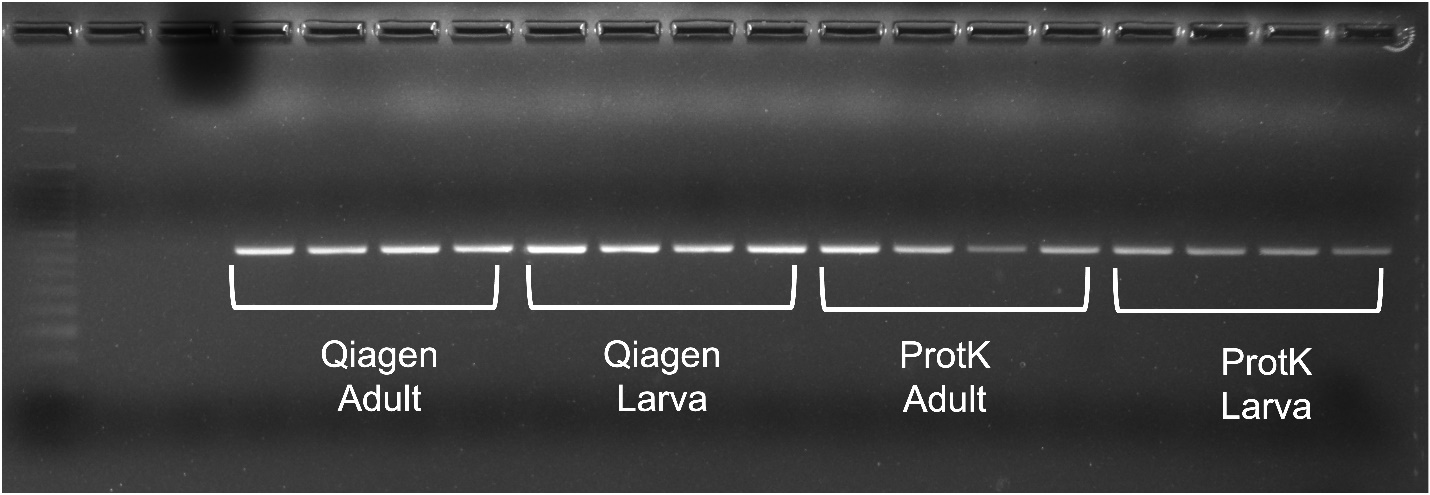


Figure S2. a) qPCR amplification plot of *Trogoderma granarium* and b) amplification plot of *T. variabile* or *T. inclusum*. Yellow curve represents the khapra beetle specific assay, and the blue curve represents the general assay.


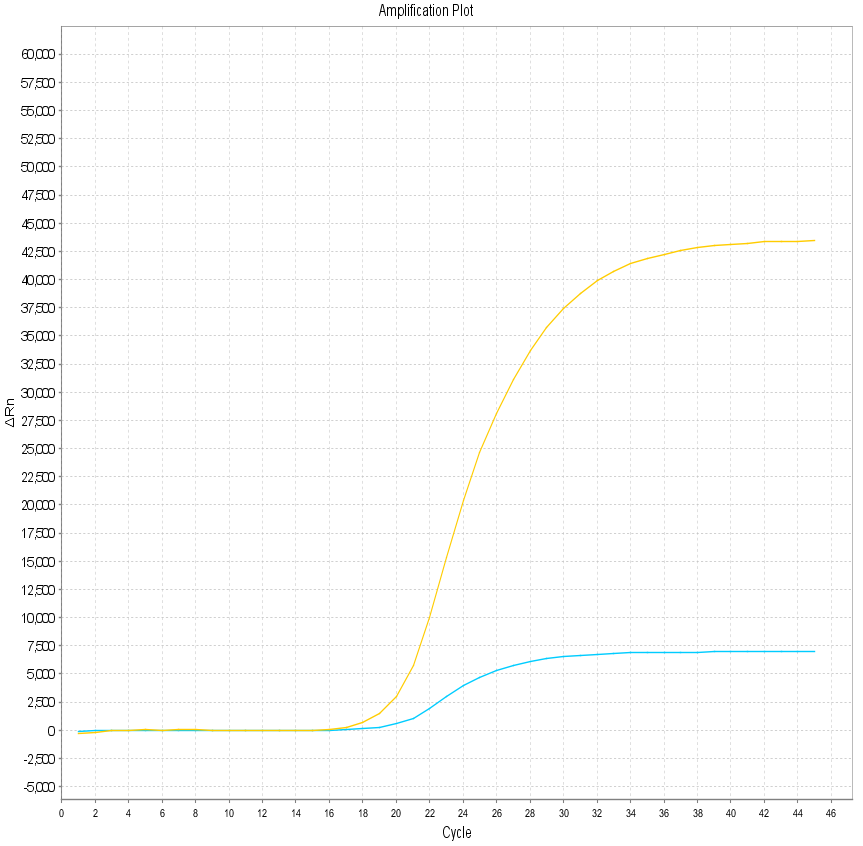


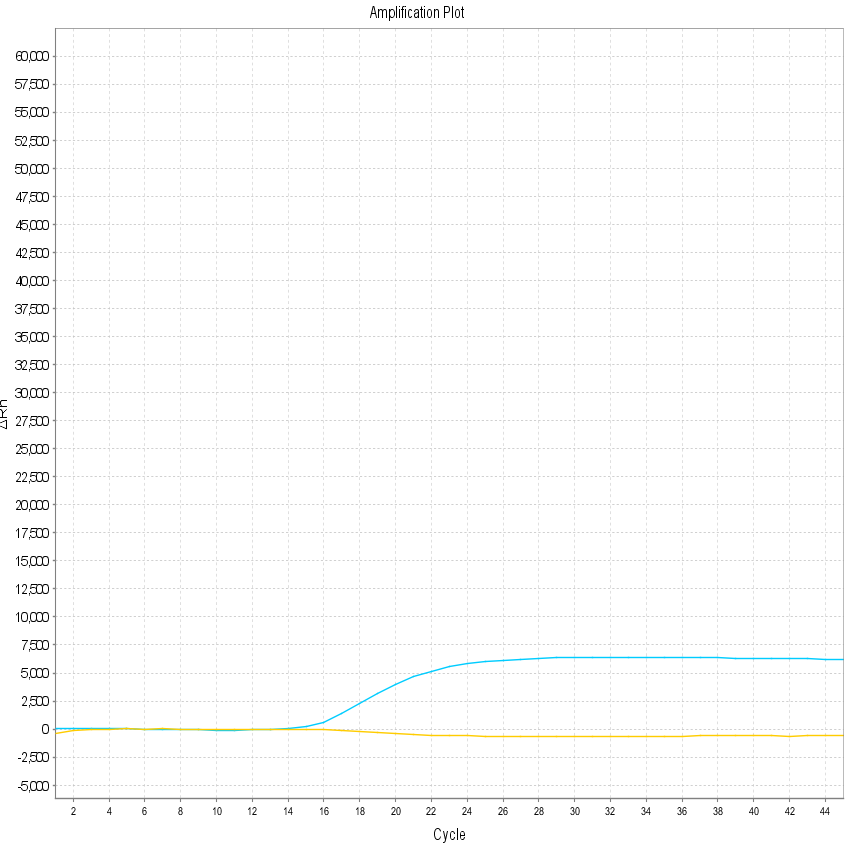


Figure S3. Standard curves of the specific and general assay running in multiplexing mode. The two regression lines nearly overlap. Red squares denote technical replicates at each dilution serial. Assay efficiency and linearity are shown below the standard curve.


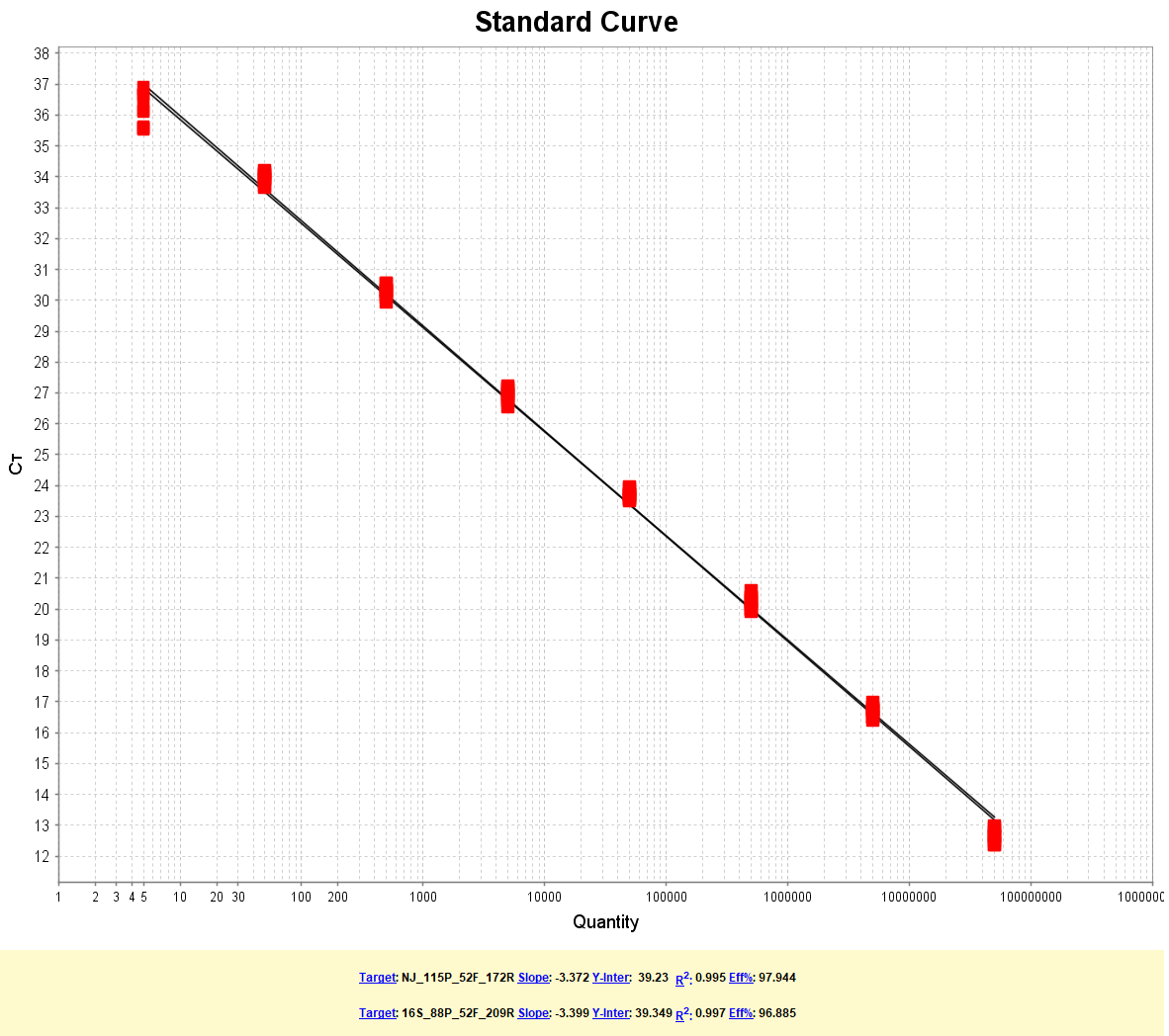


Figure S4. Standard curves of the specific (top) and general assay (bottom) running in the individual mode.


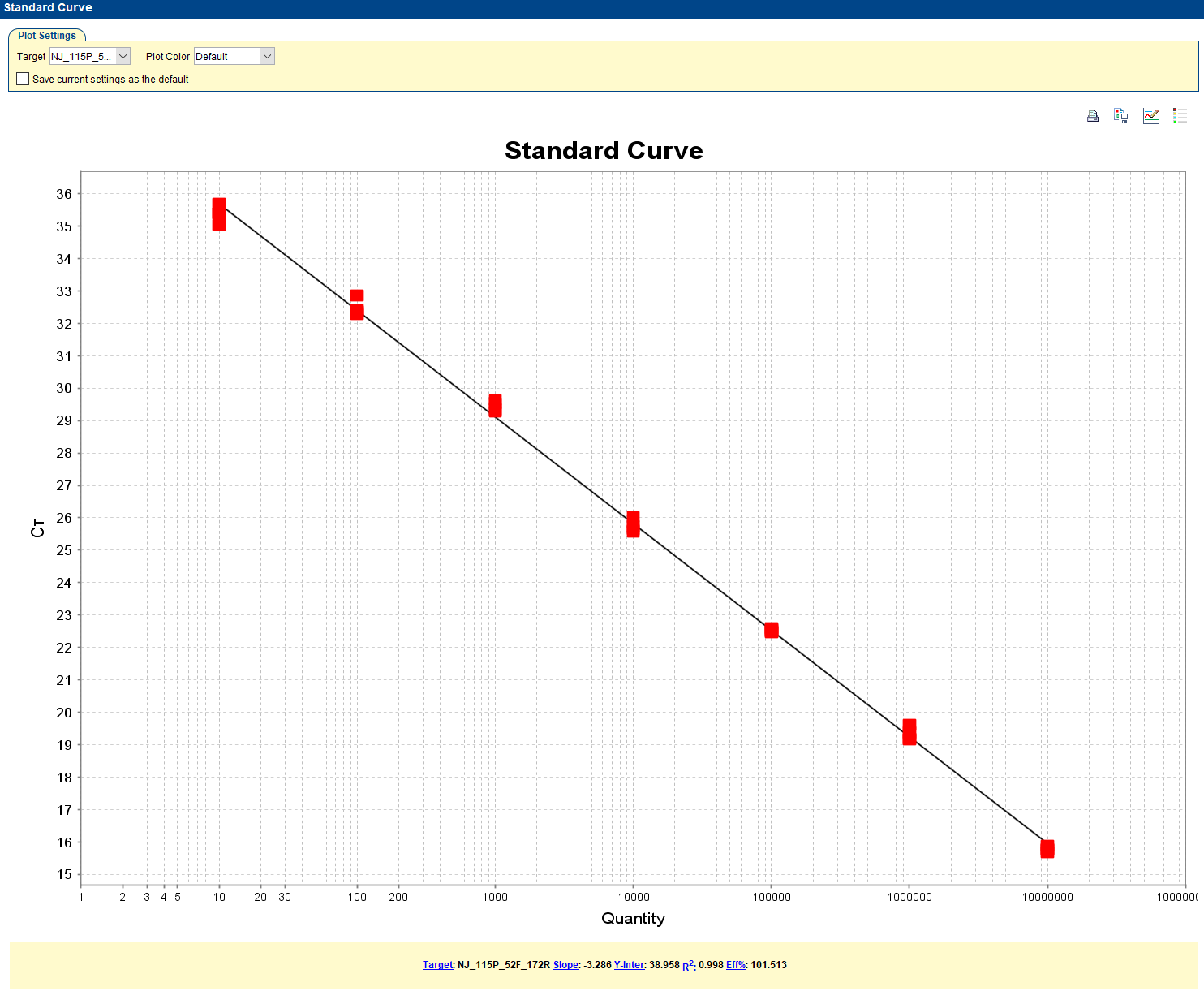


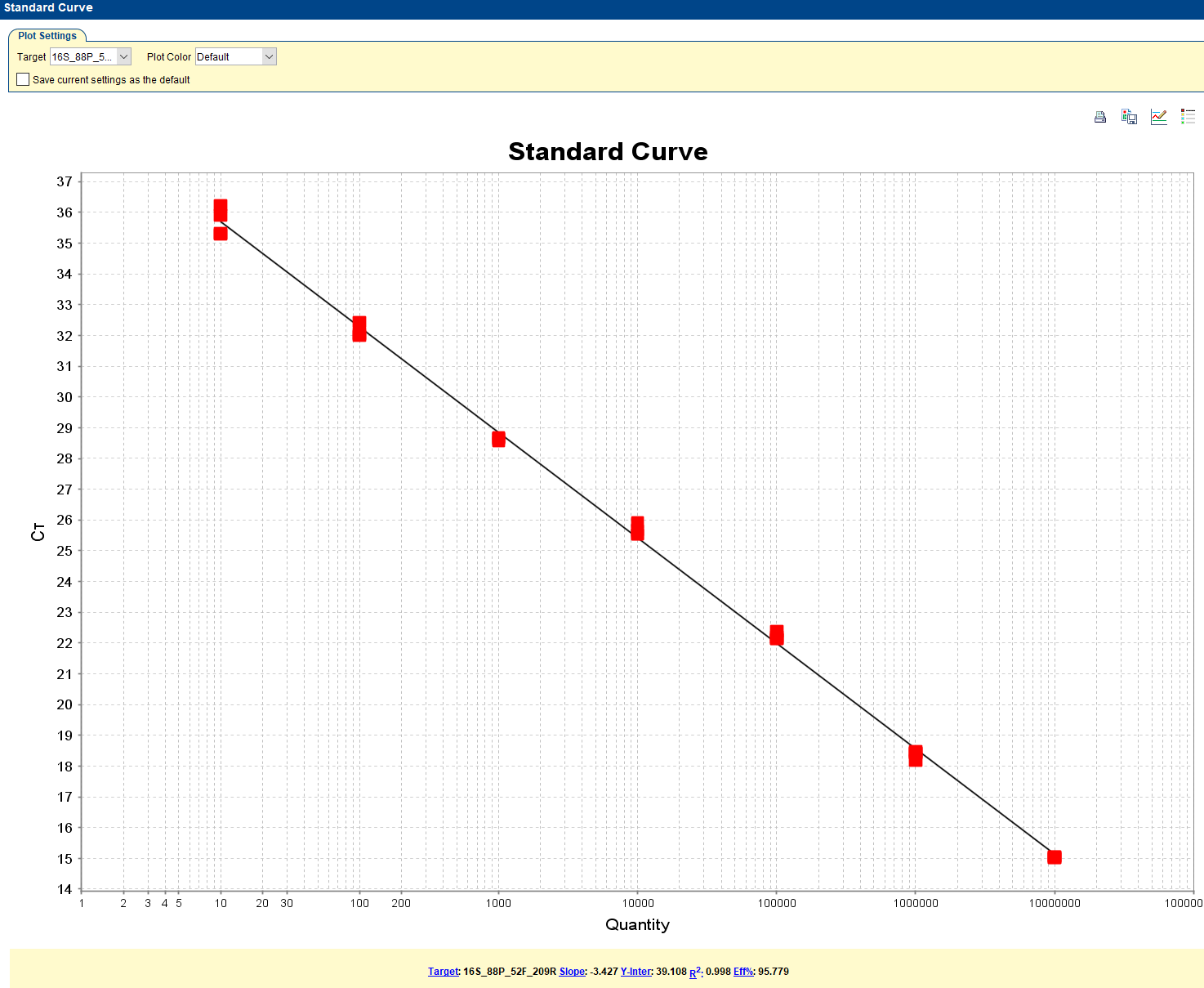

Supplement: Supplementary file 1 — Supplementary Information. [file 41598_2023_29842_MOESM1_ESM.docx]
